# The Sphagnum-Moss Gatherers

**Published:** 1916-09-23

**Authors:** 


### The Sphagnum-Moss Gatherers.

### A HOLIDAY REMINISCENCE.

A British surgeon working in a Red Cross Hospital in Italy writes: ". . . The dressings (sphagnum moss) you sent when the unit came out here proved a boon, far more than then realised, as they did not know their usage. The wounds are usually very septic, especially compound fractures, and both the moss bags and the sawdust bags have saved work and a less efficient dressing. I would be so glad to get more of both if you can send them, particularly the moss. I feel the success of many cases has been due to them. . . . I am sorry to trouble you with this extra work, but the last lot has been such a blessing."—*Scotsman.*

"Are you going for the moss to-day?" Such was the daily question of the children in the Highland village of our autumn sojourn. Indeed, so eagerly did they join in this strange new industry of gathering the sphagnum moss for the good of their country, that we feared we might appear to the uninitiated as some new version of the Pied Piper luring the children away by some uncanny charm. The most nimble of the party led the way through the glen, with its shivering aspens and birches just touched with yellow, to the peat-bog where the sphagnum grows. And how fascinating it became to draw out handfuls of the long chenille-like substance, and place it under the wide-spreading fir-tree which was selected as the first drying "depôt"! How proudly the little figures came across the moor, staggering under the weight of their dripping bundles! A few days of the glorious September sunshine soon completed the drying of the moss, and then the joy of sitting in the "depôt" or by the river-side picking out all the fir needles and other "foreign bodies," and returning with bags full of the beautiful sponge-like substance, which was to do its healing work in France, or in the nearer hospitals of our own land!

Those who were at first inclined to scoff soon showed symptoms of this strange patriotic fever, and when we visited their farm beside the standing-stones of the Druids, there we found bundles of the familiar sphagnum occupying the garden-seat in the sun, and a certain gleam in the eye of the new gatherer told its own tale.

But the day came when the holidays were at an end and you met the former little sphagnum-gatherers clad in decent tweeds and tartans, wending their way, more or less unwillingly, to school. And for the sphagnum-gatherers of a larger growth there also came an end to the time of wandering in the forests and dallying by the river. Hard it was to leave the hills with their passing purple shadows, and the yellow harvest fields, and the "vacant wine-red moor"; but the ceasing from the sphagnum industry seemed the hardest thing of all.

